# Plexin Activation Relies on an Array of Receptors

**DOI:** 10.1371/journal.pbio.1001140

**Published:** 2011-08-30

**Authors:** Stephanie Huang

**Affiliations:** Freelance Science Writer, Sunnyvale, California, United States of America

**Figure pbio-1001140-g001:**
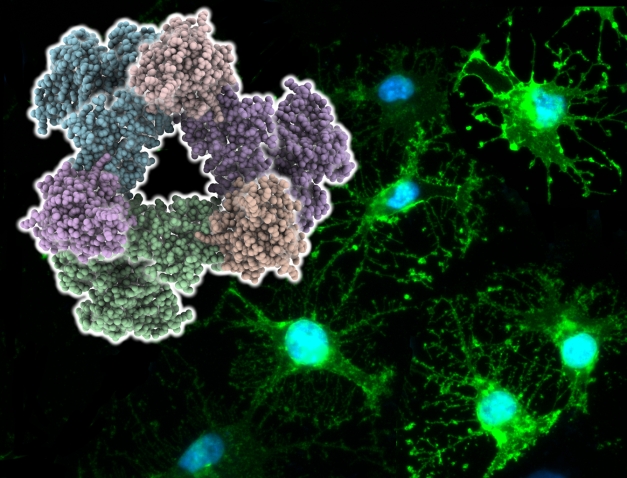
The plexin receptor mediates axon guidance, and thus proper spatial placement of neurons, during neuronal development by forming a unique trimeric complex. These individual trimeric complexes are then thought to associate to form larger arrays when bound by their extracellular semaphorin ligands.

[Fig pbio-1001140-g001]Young nerve cells put out cellular extensions called axons that must navigate through a maze of environmental cues in order to find their targets. Making the correct cellular connections is critical to building a functioning nervous system. In order to interpret these external cues, axons use receptor proteins that are directly embedded in the cell membrane. One such receptor is the plexin receptor, which recognizes and binds to an extracellular protein called semaphorin. Semaphorin is a repellent cue—when plexin detects semaphorin, it transmits signals to the inside of the nerve cell, which ultimately repels the axon away from semaphorin.

How exactly does receptor signaling work? Though receptors come in a wide variety of shapes and sizes, they share common features. Typically, some portion of the receptor sticks outside the membrane, poised to recognize proteins or small molecules that are passing by the cell, while another portion hangs inside the cell, ready to transmit a signal to internal signaling proteins. The simplest mode of receptor signaling occurs when a receptor binds to its external target and undergoes minor structural changes, changing the receptor's ability to bind to and regulate proteins inside the cell. Work from Christian Bell and colleagues, published in this issue of *PLoS Biology*, describes specifically how this works for the plexin receptor, suggesting a somewhat more complicated model in which plexin and its binding partners mediate the formation of a multiprotein array in order to activate plexin signaling.

The intracellular portion of the plexin receptor contains a region called the GTPase activating protein (GAP) domain, which binds to signaling proteins of the Ras GTPase family. Plexin binding inactivates Ras GTPases, which initiates a signaling cascade that changes the direction of axon growth. To activate the plexin GAP domain, two other proteins must simultaneously bind to plexin: semaphorin on the extracellular side and an activated member of the RhoGTPase family on the intracellular side. Thus, the plexin receptor requires binding at both ends to function properly.

A number of recent studies revealed that semaphorin binding on the extracellular side leads to the pairing of two plexin receptors. While it is known that pairing is a common mode of receptor activation, it was not clear how semaphorin- and RhoGTPase-binding activate the GAP domain. Because receptor proteins are threaded through the membrane, it is nearly impossible to study the structure of the entire protein. Instead, the researchers focused only on the intracellular region of plexin, determining the structure of this intracellular region (using human Plexin-B1) while bound to an activated member of the RhoGTPase family (Rac1*).

As expected from previous studies, the researchers' data showed that activated RhoGTPase interacts with a domain on plexin called the RhoGTPase binding domain (RBD). In fact, RhoGTPase interacts exclusively with the RBD and does not make any contacts with the GAP domain—thus, ruling out the possibility that the RhoGTPase might directly affect the GAP domain and contribute to its activation.

Interestingly, the researchers also observed a novel triplet formation—each RhoGTPase molecule appeared to interact with two molecules of the intracellular portion of plexin (Plexin-B1_cyto_). This is particularly interesting given the extracellular pairing that forms between plexin and semaphorin. The triplet revealed that activated RhoGTPase interacts with another, previously unidentified site on plexin (which the researchers refer to as site B), in addition to the RBD. The team confirmed that this site is important by showing that mutations in site B negatively impact both the binding of RhoGTPase to plexin and plexin function in the cell. Because site B is very close to the site on the GAP domain where Ras GTPases are thought to bind, it may mediate how activated RhoGTPases contribute to the activation of the GAP domain.

Bell and colleagues also identified a helix in Plexin-B1_cyto_ that can block access to site B. Based on its positioning, the helix could determine whether plexin can interact with RhoGTPases and affect overall plexin activation and signaling. In the native cellular environment, this intracellular helix would sit next to the membrane, making it possible that it plays a role in conveying signals between the extracellular and intracellular portions of plexin.

Based on their data, the researchers propose a model of plexin activation in which RhoGTPase initially binds to the RBD, and then, perhaps upon semaphorin binding and rearrangement of the intracellular helix, RhoGTPase also binds to the newly revealed site B, stabilizing the triplet complex. Taking previous studies into account, the researchers speculate that activated plexin might form an extended array, mediated by semaphorin-plexin pairs on the extracellular side and plexin-RhoGTPase triplets on the intracellular side. In such an array, GAP activity of plexin would be activated, resulting in inactivation of Ras GTPases and propagation of the resulting cellular signals.

Whether plexin-RhoGTPase triplets can actually form in the cell remains to be seen. This study, however, has provided critical new details that help fill in the molecular details of how plexin is activated by its binding partners. Because plexins and semaphorins play roles not only in axon guidance, but also in processes like angiogenesis, immune cell regulation, and tumor progression, a detailed understanding of plexin-semaphorin interaction and how it contributes to biological function will be critical for understanding the role of these proteins in these cellular processes.

**Bell CH, Aricescu AR, Jones EY, Siebold C (2011) A Dual Binding Mode for RhoGTPases in Plexin Signalling. doi:10.1371/journal.pbio.1001134**

